# Update to the Treatment of Parkinson’s Disease Based on the Gut-Brain Axis Mechanism

**DOI:** 10.3389/fnins.2022.878239

**Published:** 2022-07-06

**Authors:** Xiaohui Sun, Li Xue, Zechen Wang, Anmu Xie

**Affiliations:** ^1^Department of Neurology, The Affiliated Hospital of Qingdao University, Qingdao, China; ^2^Recording Room, The Affiliated Hospital of Qingdao University, Qingdao, China

**Keywords:** Parkinson’s disease, gut, gut-brain axis, constipation, mechanisms, treatment, microbiota

## Abstract

Gastrointestinal (GI) symptoms represented by constipation were significant non-motor symptoms of Parkinson’s disease (PD) and were considered early manifestations and aggravating factors of the disease. This paper reviewed the research progress of the mechanism of the gut-brain axis (GBA) in PD and discussed the roles of α-synuclein, gut microbiota, immune inflammation, neuroendocrine, mitochondrial autophagy, and environmental toxins in the mechanism of the GBA in PD. Treatment of PD based on the GBA theory has also been discussed, including (1) dietary therapy, such as probiotics, vitamin therapy, Mediterranean diet, and low-calorie diet, (2) exercise therapy, (3) drug therapy, including antibiotics; GI peptides; GI motility agents, and (4) fecal flora transplantation can improve the flora. (5) Vagotomy and appendectomy were associated but not recommended.

## Highlights

– Neuroendocrinology plays an important role in the gut-brain axis of Parkinson’s disease.
– Familial aggregation of Parkinson’s disease may be associated with family *Helicobacter pylori* infection.
– Dietary restriction and a ketogenic diet reduce the risk of Parkinson’s disease.
– Gastrointestinal motility drugs and diabetes drugs may also beneficial for Parkinson’s disease.

## Introduction

Parkinson’s disease (PD) is a slowly progressive neurodegenerative disease. Its pathology is characterized by the death of dopaminergic neurons in the substantia nigra (SN) and the formation of Lewy bodies by abnormal aggregation of alpha-synuclein (α-syn) (Kalia and Lang, 2015). The typical clinical manifestations of PD include tremor, myotonia, loss of movement, and postural disorders, as well as non-motor symptoms such as depression, anxiety, sensory disorders, sleep disorders, constipation, memory loss, anosmia, and dementia (Poewe et al., 2017). Neuropathology has shown that the anterior olfactory nucleus and several nuclei in the brainstem were affected before the SN of the midbrain is damaged (Braak et al., 2003). Depending on this hypothesis, the non-motor symptoms occur several years earlier than motor dysfunction development. Therefore, studying the non-motor symptoms of prodromal PD is beneficial to improve the early and correct diagnosis of PD.

Parkinson’s disease patients are under a variety of gastrointestinal (GI) manifestations, including salivation, dysphagia, delayed gastric emptying, constipation, and anorectal dysfunction (including urinary incontinence). While these symptoms were not fatal, they can seriously impair the quality of life in PD patients. On the other hand, GI dysfunction can affect pharmacodynamics, lead to fluctuations in PD symptoms, and further lead to disability, as reflected in reduced benefits per unit dose of levodopa. Fortunately, these symptoms can be observed 20 years before the diagnosis of PD (Stirpe et al., 2016). If these symptoms can be detected, diagnosed, and intervened early, it may delay or even reverse the onset of PD. Constipation is among the most prominent and disabling manifestations of lower GI dysfunction in PD. Nearly 80% of Parkinson’s patients have constipation symptoms (according to the Rome III criteria) (Yu et al., 2018), and can precede the development of somatic motor symptoms of PD for several years, maybe even a decade or more (Liddle, 2018). Therefore, constipation has been incorporated into the movement disorder society (MDS) diagnostic criteria of prodromal PD (Heinzel et al., 2019).

**FIGURE 1:**
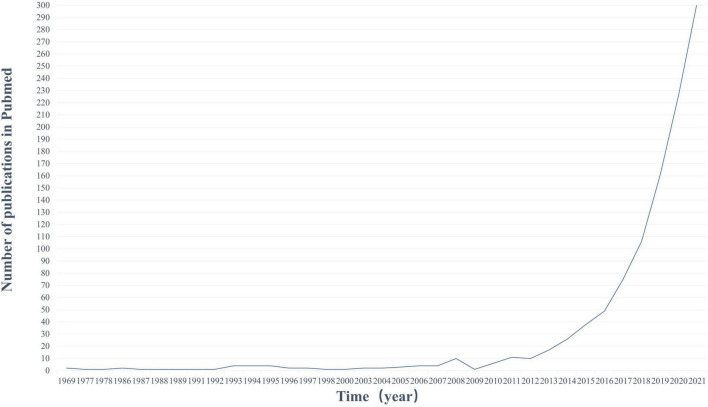
Publication trends on PubMed (1969–2020). Number of publications in PubMed per year using keywords (gut and PD). The number of publications in this area of research is increasing rapidly.

Based on these findings, scientists’ interest in the association between gut and PD has risen steadily (Figure 1). Exploring whether and how there is a link between the GI tract and PD is the subject of ongoing research and discussion in the industry. We have analyzed and discussed the roles of α-synuclein, GI flora, immune inflammation, neuroendocrine, and mitochondrial autophagy in the gut-brain axis (GBA) in PD. On this basis, a host of promising ideas and methods for the treatment of PD have been seen. Increased dietary intake of probiotics, omega-3, short-chain fatty acids (SCFA), vitamins, a Mediterranean diet, as well as a low-calorie intermittent diet, and moderate exercise were thought to play a role in the development of PD. Eradication of *Helicobacter pylori* (*Hp*), GLP-1 receptor agonists, and antibiotics may also have potential therapeutic effects on PD. Vagotomy is controversial, although this is evidence to reduce the risk of PD through the GBA mechanism, we do not recommend it as a preventive treatment for PD. Fecal microbiota transplantation (FMT) has also been suggested to regulate the onset of PD by interfering with the gut microbiota.

## Materials and Methods

The contents of this paper were determined through the retrieval and selection of the following databases in the past 5 years: PubMed, Google Scholar, Scopus, and Embase. Searching was made in two steps. Firstly, using a combination of keywords: “microbiota,” “gut,” “gut-brain axis,” and “Parkinson’s disease.” Secondly, terms such as “constipation,” “Neuroendocrine,” “*H. pylori,”* “brain gut peptides,” “immunity,” “inflammation,” “omega 3,” “diet,” and “fecal transplantation” were added to the retrieval strategy to evaluate the roles of different factors in the enteric-brain axis, respectively. In addition, the bibliography of each selected article has been revised to include other relevant articles.

Statistical analysis uses Stata 16.0, which is mainly used to calculate OR (95% CI) and to map forests to assess the relationship between Hp infection and PD risk. A two-tailed *P* < 0.05 was defined as statistically significant.

## Gut-Brain Axis Pathogenesis

### α-Synuclein

The autopsy confirmed that PD patients not only had an abnormal accumulation of α-syn in the brain, but also exist in GBA, perhaps even earlier (Fitzgerald et al., 2019). The expression and misfolding of α-syn can be seen at the gut level can be observed 20 years before PD diagnosis (Stokholm et al., 2016; Zhu et al., 2017). It can be speculated that α-syn accumulated in the intestine in the early stage of PD, even before the first motor symptoms appeared (Braak et al., 2004).

Animal experiments have confirmed that α-syn can be transported to the brain through the blood-brain barrier (BBB) (Holmqvist et al., 2014; Kim et al., 2019). Truncal vagotomy and α-syn deficiency reduce α-syn associated neurodegeneration, which is evidence of α-syn spreading from the gut to the brain (Svensson et al., 2015).

However, the deposition of α-syn in the mucosal enteric nervous system (ENS) seems to have nothing to do with the functional impairment of the affected gut segment (Lee et al., 2018). Therefore, α-syn may only be the pathological origin or manifestation of PD, leading to overall GI symptoms through the nervous system, rather than direct GI damage.

### Gut Microbiota

The GI tract is the part with the highest content of microorganisms in the human body (Gilbert et al., 2018), and everyone has their own unique GI flora structure (Karl et al., 2018). The gut microbiota is now increasingly recognized as a potential participant in the pathogenesis of PD (Lubomski et al., 2019). The imbalance of GI flora can lead to GI dysfunction, and this imbalance may also be the pathogenesis of PD itself. As shown in Table 1, the changes in gut microbiota in PD patients were determined through multiple studies (Keshavarzian et al., 2015; Scheperjans et al., 2015; Unger et al., 2016; Heintz-Buschart et al., 2018; Barichella et al., 2019; Lubomski et al., 2020; Ren et al., 2020).

**TABLE 1:** Changes in gut microbiota in Parkinson’s disease (PD) patients compared to normal controls.

Phylum	Class	Order	Family	Genus
**Increased gut microbiota in PD**				
Actinobacteria	Actinomycetia	Bifidobacteriales	*Bifidobacteriaceae*	*Bifidobacterium*
		Coriobacteriia	*Coriobacteriales*	*Coriobacteriaceae*
				*Collinsella*
Bacteroidetes	Bacteroidia	Bacteroidales	*Barnesiellaceae*	*Barnesiella*
			*Odoribacteraceae*	*Butyricimonas*
				*Odoribacter*
			*Porphyromonadaceae*	
			*Rikenellaceae*	*Alistipes*
Firmicutes	Bacilli	Bacillales	*Bacillaceae*	*Bacillus*
		Lactobacillales	*Enterococcaceae*	
			*Lactobacillaceae*	*Lactobacillus*
			*Streptococcaceae*	*Streptococcus*
	Clostridia	Eubacteriales	*Christensenellaceae*	
			*Clostridiaceae*	*Hungatella*
			*Oscillospiraceae*	*Anaerotruncus*
				*Hydrogenoanaerobacterium*
			*Ruminococcaceae*	
	Erysipelotrichia	Erysipelotrichales	*Erysipelotrichaceae*	*Allobaculum*
				*Erysipelatoclostridium*
	Negativicutes	Veillonellales	*Veillonellaceae*	*Megasphaera*
		Acidaminococcales	*Acidaminococcaceae*	*Acidaminococcus*
Proteobacteria	Betaproteobacteria	Burkholderiales	*Burkholderiaceae*	*Ralstonia*
	Gammaproteobacteria	Enterobacterales	*Enterobacteriaceae*	*Enterobacter*
				*Escherichia*
		Moraxellales	*Moraxellaceae*	*Acinetobacter*
Verrucomicrobia	Verrucomicrobiae	Verrucomicrobiales	*Akkermansiaceae*	*Akkermansia*
			*Verrucomicrobiaceae*	
**Declined gut microbiota in PD**				
Bacteroidetes	Bacteroidia	Bacteroidales	*Prevotellaceae*	*Prevotella*
Firmicutes	Clostridia	Eubacteriales	*Clostridiaceae*	*Clostridium*
			*Eubacteriaceae*	*Eubacterium*
			*Lachnospiraceae*	*Blautia*
				*Dorea*
				*Roseburia*
				*Coprococcus*
				*Fusicatenibacter*
				*Lachnospira*
			*Oscillospiraceae*	*Faecalibacterium*
				*Ruminococcus*
			*Peptostreptococcaceae*	*Terrisporobacter*
Proteobacteria	Gammaproteobacteria	Pasteurellales	*Pasteurellaceae*	*Haemophilus*

The PD microbiota was characterized by reduced carbohydrate fermentation and butyric acid synthesis and increased proteolytic fermentation and production of harmful amino acid metabolites, including para-cresol and phenylacetylglutamine. These taxonomic changes and the elevation of proteolytic metabolites were also strongly associated with stool consistency and constipation in patients (Cirstea et al., 2020). According to the functional characteristics of these bacteria, dysregulation of gut microorganisms can lead to increased intestinal mucosal permeability, inflammation, the impaired balance of SCFA, and/or oxidative stress, which may trigger the accumulation of α-syn (Dalile et al., 2019). This is consistent with reports of intestinal leakage and reduced levels of SCFA and lipopolysaccharide (LPS)-binding proteins in PD patients (Hasegawa et al., 2015; Unger et al., 2016). However, the levels of serum diamine oxidase, a marker for intestinal mucosal integrity, remained unchanged in PD (Hasegawa et al., 2015).

Changes in the gut microbiota may have more impact than we thought and different microbiota affect different biological functions. Our microbial community depends on multiple variables, including stress, toxic substances, antibiotics, environmental pathogens, infections, physical activity, diet, pollutants, noise, lifestyle, and the environment (Karl et al., 2018). This suggested that the GBA is bidirectional.

#### Probiotics

Probiotics were defined as “Living microorganisms that are beneficial to the host when sufficient quantities are given,” referring to microbes that have beneficial functions in our bodies. Interestingly, changes in GI flora in PD patients showed a decrease in some probiotics, such as *Faecalibacterium* and *Roseberia*, but increased levels of *Akkermansia, Bifidobacteria*, and *Lactobacillus*. Perhaps supplementing those reduced probiotics can help treat PD. People with a high abundance of *Prevotellaceae* were very unlikely to have PD (Scheperjans et al., 2015).

The benefits of probiotics to animals and humans are numerous. They can effectively improve the GI ecosystem, maintain the integrity of intestinal intima, contribute to the formation of GI flora and balance the *pH* value of the body (Liu et al., 2015). Probiotics can reduce intestinal pH by fermenting dietary fiber and resistant starch to produce SCFAs. The neuroprotective effect of a probiotic mixture containing *Bifidobacterium animalis lactis, Lactobacillus rhamnosus GG*, and *Lactobacillus acidophilus* was evaluated in an animal model of PD, and the probiotic now partially ameliorates neurodegeneration by increasing butyrate levels (Srivastav et al., 2019; Sun et al., 2021). Bacteria such as *Lactobacillus* and *Enterococcus* will metabolize levodopa into dopamine through tyrosine decarboxylase, and these bacteria will also increase in the intestines of PD patients. If we reduce these bacteria in the intestine of PD patients, it is possible to reduce the peripheral metabolic loss of levodopa and improve its utilization in the CNS, which may provide an idea for the individualized treatment of PD (Maini Rekdal et al., 2019). Probiotics can promote the production of brain-derived neurotrophic factor (BDNF), which can regulate brain function, reduce anxiety and depression, ameliorate cognitive dysfunction, maintain and promote the development, differentiation, growth, and regeneration of 5-hydroxytryptamine (5-HT) and dopaminergic neurons (Ruiz-Gonzalez et al., 2021). Probiotics can influence the production of neurochemicals and reduce neurodegeneration, thus effectively improving functions related to mental illness and memory skills (Wang et al., 2016; Leta et al., 2021).

Probiotics also increase glucagon-like peptide-1 (GLP-1) secretion and decrease insulin resistance to increase glucose metabolism (Leta et al., 2021). Probiotics produce antimicrobial substances called bacteriocins that act like antibiotics, regulate immunity and control inflammation, block the spread and invasion of pathogen bacteria, and prevent the presence of inflammatory compounds in the brain. For example, *Lactobacillus plantarum* reduces gut permeability and inflammatory levels (Mallikarjuna et al., 2016). Probiotics reduce interleukin-6 and tumor necrosis factor-alpha (TNF-α) and C-reactive protein (CRP) levels to reduce peripheral and central inflammation (Leta et al., 2021). Probiotics also reduce the levels of LPS, whose physiological effects were expressed by toll-like receptor 4 (TLR4). TLR4 exists on the surface of the cell membrane of host cells, and its mediated gut dysfunction may be related to enteric and central inflammation of PD (Perez-Pardo et al., 2019). Oxidative stress is another factor in the degeneration of dopaminergic neurons in PD. Probiotics have the highest capacity to produce potentially antioxidant molecules (Leta et al., 2021).

Probiotics can promote the absorption of food and improve the bioavailability of certain nutrients, such as A, C, and K vitamins and those of the B group (Liu et al., 2015). Vitamins may be cofactors in the catecholamine biosynthetic pathways. *Lactobacillus lesei* has been shown to increase the excitability of intermuscular neurons in rats, affecting enteric afferent nerve and GBA interactions. Probiotic-mediated modulation of microbial-gut-brain interactions is considered a potential new therapeutic tool for the treatment of gut motility disorders, reducing overall PD motility symptoms and non-motility symptoms such as constipation (Leta et al., 2021; Table 2).

**TABLE 2:** Effect of probiotics on Parkinson’s disease (PD).

Country	References	Study	Probiotic feeding forms	Placebo	Changes of the probiotics group
Itlay	Barichella et al., 2016	Randomized, double-blind, controlled trial	Fermented milk containing multiple probiotic strains and prebiotic fiber (250 billion CFU)	A pasteurized, fermented, fiber-free milk	**Increases** the frequency of CBMs
Iran	Borzabadi et al., 2018	Randomized, Double-blind, Placebo-Controlled Trial	8 × 10^9^ CFU/day probiotic supplements capsules	Capsules with similar packaging to probiotic supplements	**Improved** gene expression of IL-1, IL-8, TNF-α, TGF-β, and PPAR-γ; **no affect** of gene expression of VEGF and LDLR, and biomarkers of inflammation and oxidative stress.
Iran	Tamtaji et al., 2019	Randomized, Double-blind, Placebo- Controlled Trial	8 × 10^9^ CFU/day probiotic supplements capsules	Capsules with similar packaging to probiotic supplements	**Useful impact** on MDS-UPDRS, hs-CRP, GSH, MDA, insulin metabolism
Malaysia	Ibrahim et al., 2020	Randomized controlled trial	multi-strain probiotic (*Lactobacillus* sp. and *Bifidobacterium* sp. at 30 × 10^9^ CFU) with fructooligosaccharide	Fermented milk	**Improved** bowel opening frequency and whole gut transit time
Malaysia	Tan et al., 2021	Randomized Placebo-Controlled Trial	multi-strain probiotics capsules (10 billion CFU per dose)	Capsules with similar packaging to probiotic supplements	**Improved** the number of SBM per week in PD patients with constipation.

*BM, bowel movement; CBM, complete bowel movement; SBM, spontaneous bowel movements.*

#### Harmful Bacteria: *Helicobacter pylori*

Gut bacteria are interdependent and symbiotic. A series of problems caused by gut dysbiosis in PD patients have been described above. The following sections take *Hp* as an example to discuss the relationship between harmful bacteria in the gut of PD patients and the progression of PD.

About half of the earth’s population carries *H. pylori* (Alexander et al., 2021). As the only bacteria that can survive in the human stomach for a long time, it is one of the culprits of digestive diseases such as chronic gastritis, peptic ulcer, and gastric cancer (Dobbs et al., 2000a). Triple or quadruple therapy of proton pump inhibitor (PPI), bismuth, and 1-2 antibiotics is commonly used clinically to eradicate *H. pylori*. *Hp* can be absorbed by dopamine, while PD patients lack dopamine, which may be one of the reasons why the infection rate of *Hp* in PD patients is higher than that in normal people (Charlett et al., 1999; Table 3). Dopaminergic drugs can inhibit the growth of *Hp* and benefit PD patients. Dopamine antagonists can cause experimental ulcers (Dobbs et al., 2000a). The higher positive rate of serum *Hp* in PD patients may be due to host susceptibility, or conversely, infection with specific *Hp* strains reduces dopaminergic status (Dobbs et al., 2000a).

**TABLE 3:** Effect of radical cure of *Helicobacter pylori* on Parkinson’s disease (PD).

Country	References	Study	Method of Hp detection	Hp (+) in the PD patients group	Hp (+) in the control group	OR(95%CI)
United Kingdom	Charlett et al., 1999	Case-control	ElISA	23/33	31/78	3.49(1. 46, 8.32)
United Kingdom	Dobbs et al., 2000b	Case-control	ElISA	25/58	43/136	1.64 (0.87, 3.09)
Krean	Lee et al., 2008	nRCT	^13^C-urea breath test	35/65	–	–
United Kingdom	Charlett et al., 2009	Cross-sectional	ElISA	57/120	77/196	1.40 (0.88, 2.21)
Denmark	Nielsen et al., 2012	Case-control	Prescriptions for Hp eradication drugs	138/4484	505/22416	1.38(1.14, 1.67)
United Kingdom	Blaecher et al., 2013	Case-control	RT-PCR	17/60	42/256	2.01 (1.05, 3.87)
Malaysia	Nafisah et al., 2013	Cross-sectional	^13^C-urea breath test	14/29	5/23	3.36 (0.98, 11.49)
China	Bu et al., 2015	Case-control	ElISA	60/131	44/141	1.86 (1.14, 3.06)
Malaysia	Tan et al., 2015	Case- cohort	^13^C-urea breath test	33/102	–	–
Greece	Tsolaki et al., 2015	Case-control	Gastroscopy and histologic examination	6/9	14/31	2.43 (0.51, 11.51)
India	Mridula et al., 2017	Prospective case cohort	ElISA	18/36	–	–
Greece	Efthymiou et al., 2017	Case-control	ElISA	14/39	33/68	0.59 (0.26, 1.33)

*Helicobacter pylori* aggravates the symptoms of PD by affecting the absorption of levodopa, the main drug for the treatment of PD. PD patients with *HP* infection were less responsive to drugs, have more severe motor symptoms, and motor complications were easy to occur (Zhong et al., 2022). Eradication of *Hp* infection in PD patients with antibiotics can improve GI symptoms, facilitate the absorption of levodopa, increase daily “on” time, reduce motor fluctuations, and ameliorate motor function to a certain extent (Lee et al., 2008; Çamcı and Oğuz, 2016; Bai and Li, 2021; Lolekha et al., 2021). However, according to the latest study by Tan et al. in Malaysia, *Hp* eradication has no significant improvement in motor symptoms, non-motor symptoms, or quality of life in PD patients (Tan et al., 2020).

**FIGURE 2:**
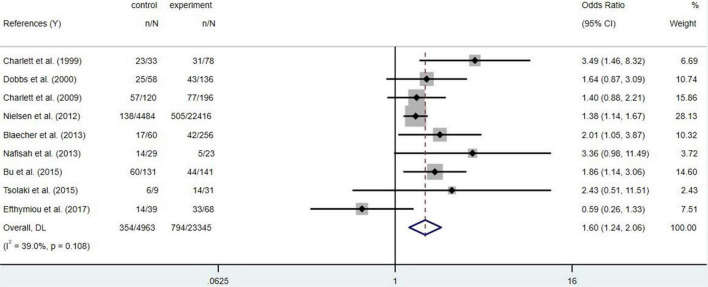
Forest map of *Helicobacter pylori* (*Hp*) infection and the Parkinson’s disease (PD) risk.

To sum up, *Hp* infection appears to be associated with PD risk, but the available evidence found no statistical difference (Figure 2, *p* = 0.108). Family-gathered *Hp* may increase the risk of familial PD, which may be mistaken for hereditary PD. Eradication of *Hp* infection is not associated with a reduced risk of PD, but may improve the bioavailability and therapeutic efficacy of levodopa (Nyholm and Hellström, 2021). Further studies are needed to determine whether *Hp* eradication can optimize the prognosis of PD.

### Neuroendocrine

The brain communicates with the viscera through multiple parallel pathways, such as the autonomic nervous system (ANS), the hypothalamic-pituitary-adrenal axis (HPA), the sympathetic-adrenal axis, and the descending monoaminergic pathway (Mayer, 2011).

#### Nervous Systems

##### Enteric Nervous Systems

The ENS is the largest cluster of neurons and glial cells outside the CNS (de Weerth, 2017). The ENS is huge and complex, which can control and regulate GI function independently of the CNS. A large number of neurons buried in the GI wall constitute a multitude of GI ganglia, also known as nerve plexus, together with the surrounding capillaries to form a “blood-intestinal plexus barrier” similar to the “blood-brain barrier.” An intact ENS is essential to health, and ENS dysfunction is often associated with digestive diseases (Rao and Gershon, 2016).

Mucosal biopsy samples show that abnormally folded α-syn aggregates form Lewy bodies and neurites in the ENS of PD patients before clinical diagnosis or untreated (Travagli et al., 2020). Studies focusing on Lewy body pathology in the ENS have found α-syn in the ENS of almost every PD patient. As previously mentioned, large abnormal aggregation of α-syn was also found in the GI of PD patients. Therefore, based on the discovery of Lewy bodies in the GI before the symptoms of PD, it is reasonable to infer that α-syn is introduced from the GI to the brain through ENS, ultimately leading to the manifestation of PD (Rota et al., 2019). In PD patients, there is no denying that α-syn bi-directional transport may aggravate the GI symptoms of PD due to the presence of ENS. The interaction between the ENS and CNS is often described as the GBA.

##### Autonomic Nervous System

Abundant neuronal loss and pathological aggregation of α-syn were observed in both sympathetic and parasympathetic nerves of PD patients (Uchihara and Giasson, 2016). The significant involvement of the GI tract and peripheral ANS is one of the characteristics that distinguish PD from other neurodegenerative diseases. It has been proposed that the α-syn aggregation in some PD patients originated from the unmyelinated, hyperbranched axons, and terminals of the peripheral autonomic nerve terminals and then spread centripetally by axonal transport to the central nervous system (CNS).

**FIGURE 3:**
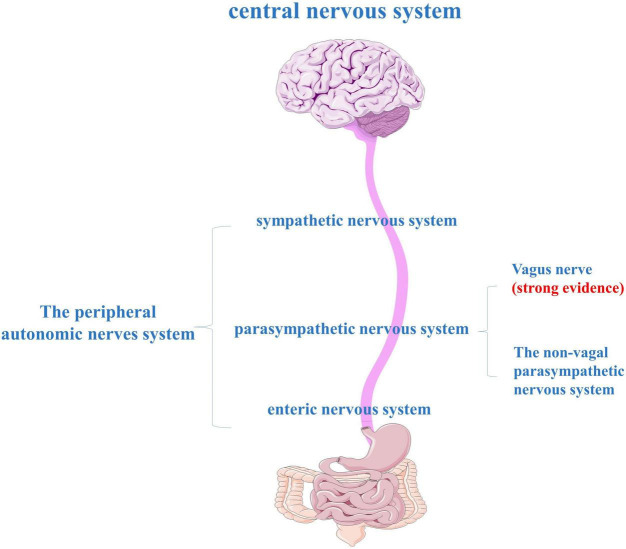
The peripheral autonomic nervous system (ANS) consists of three main parts: the sympathetic nervous system, the parasympathetic nervous system, and the enteric nervous system (ENS).

The parasympathetic component of the vagus is the main component of the parasympathetic nervous system and plays a prominent role in the regulation of GI motor function (Chen et al., 2020). The vagus nerve is one of the largest nerves connecting the GI tract and the brain, and it is a crucial pathway for the transmission of β-syn between the periphery and the brain (Figure 3). In animal experiments, α-syn can spread from the duodenum to the brain stem and then from the brain stem to the stomach *via* the vagus nerve (Ulusoy et al., 2017; Uemura et al., 2018; Kim et al., 2019; Van Den Berge et al., 2019). Although the level of α-syn used in the above was much higher than in PD, it may not accurately mimic human pathophysiology, but the existence of this pathway had been explored to some extent. The dual-hit hypothesis suggests that α-syn pathology initially forms in the olfactory bulb and the autonomic nerve endings of the GI mucosa. Then transported prion-like centrally from the ENS to the DMV *via* retrograde axons of the vagus nerve, and from there to more rostral areas of the CNS (Braak et al., 2003; Borghammer, 2018; Kujawska and Jodynis-Liebert, 2018). It can be hypothesized that gut neuropathogens ingested in the environment may induce or accelerate the progression of PD by entering the CNS through the vagus nerve (Larroya-García et al., 2019; Travagli et al., 2020). About half of the neurons in the dorsal nucleus of the vagus nerve in PD patients were lost during the disease (Braak et al., 2003). Ultrasound also found a reduction in the diameter of the vagus nerve, indicating that the degeneration of the ANS may play a key role in the pathogenesis of PD, especially the vagus, which may be a potential target for therapy (Orimo et al., 2018).

As an important surgical procedure for peptic ulcer disease, vagotomy has also been found to reduce the risk of PD, especially full truncal vagotomy (Svensson et al., 2015; Liu et al., 2017; Girard-Madoux et al., 2018). This showed that the vagus nerve is not only involved in the transmission of PD but also plays a key role in the pathogenesis of PD, supporting the view that PD is closely related to the gut. However, vagotomy will not be performed in the early clinical stages of PD. This is because although it can reduce the risk of developing PD, it can also lead to serious complications such as dysphagia, vomiting, and diarrhea, which can have incalculable side effects on the intestines, brain, and other organs. Therefore, effective screening of people at risk for PD, and thus necessary early intervention, is crucial (Rahimpour et al., 2022).

##### Enteric Glial Cells

The enteric glial dysfunction in the colon of PD patients in association with the early appearance of Lewy pathology (Lewy bodies and neuritis) in neurodegeneration areas of the ENS (Shannon et al., 2012). Some pathological gut diseases associated with impaired intestinal barrier permeability may trigger EGCs dysfunction and transition to a pro-inflammatory phenotype (Benvenuti et al., 2020). Chronic constipation can impair the activity of enteric glial cells (EGC), cause localized enteritis, and changes in enteric neuronal activity, resulting in early deposition of extracellular α-syn and typical PD-related gut motor dysfunction. In addition, the pro-inflammatory markers related to glial cells in the colon of PD patients were increased, such as GFAP, SOX-10, IL-6, IL-1β, and TNF- α (Clairembault et al., 2015). This reactive glial hyperplasia destroys the integrity of the intestinal barrier and the neuroinflammatory response rises to the CNS through the vagus nerve or glial cell Cx-43. This may be related to neuroinflammation associated with PD and pathological changes associated with the ENS (Benvenuti et al., 2020). A possible turning point in the treatment of PD in the development of a new EGC-targeted therapy to regulate the activity of EGC in the early stages of the disease (Seguella et al., 2019).

Astrocytes are the most abundant glial cells in the CNS. In the ENS, astrocytes like EGCs play an important role in protecting enteric neurons by secreting neurotrophic factors and antioxidant factors, and are also potential neuroprotective factors in PD (Isooka et al., 2021). Like glial cell line-derived neurotrophic factor (GDNF) mediated by lentiviral vectors, the antioxidant molecules glutathione (GSH) and metallothionein (MT)-1,2 (Chung et al., 2004; Dowd et al., 2005; Steele et al., 2013). Furthermore, targeting 5-HT1A receptors on astrocytes may be a potential neuroprotective strategy for PD. Rotigotine, for example, upregates the expression of MT-1 and MT-2 by activating the astrocyte 5-HT1A receptor, and mirtazapine also has a similar effect (Miyazaki and Asanuma, 2016; Isooka et al., 2021).

##### Neurotransmitters

Neurotransmitters can play an important role in regulating GBA, including adenosine, acetylcholine (Ach), dopamine (DA), epinephrine (E), glutamate, histamine, norepinephrine (NE), serotonin (5-HT), and γ-aminobutyric acid (GABA), The levels of neurotransmitters in the striatum of PD patients changed as follows: DA, GABA, adenosine decreased; glutamate and Ach increased (Jamwal and Kumar, 2019).

Dopamine is the most ubiquitous catecholaminergic neurotransmitter that regulates movement, cognition, emotion, and positive reinforcement (Luo and Roth, 2000). The decrease of dopamine level is accountable for the regular manifestation and progression of the signs and symptoms of PD.

5-hydroxytryptamine (5-HT), also known as serotonin, is a critical signaling regulator that can modulate complex physiological functions such as gastric secretion, cognitive function, and body temperature. 95% of serotonin is synthesized in the intestine, in which gut microorganisms play an important role.

Generally, neurons are activated by glutamate and inhibited by GABA. The abnormal changes of these two activation systems are one of the pathophysiological bases of neurological diseases. Glutamate is the principal excitatory neurotransmitter in the basal ganglia of the midbrain, which plays a central role in mediating excitatory neurotransmission. The normal concentration of glutamate is necessary for the physiological function of the brain, but when it exceeds normal, it will cause the death of excitatory neurons and produce neurotoxic or excitotoxic events (Dong et al., 2009). In neurodegenerative diseases, the excitotoxicity caused by glutamate causes the death of a large number of neurons (Jamwal and Kumar, 2019). Neurotransmitter alterations in direct and indirect nigrostriatal pathways occurring in PD were known to involve glutamatergic hyperactivity. Furthermore, clinical and preclinical evidence found that the mRNA and protein levels of GABA B receptors decreased in globus pallidus external (GPe), and increased in globus pallidus internal (GPi) (Calon et al., 2003).

The pre and post-ganglionic parasympathetic neurons are cholinergic, accounting for 70% of the ENS. M4 receptor in striatum mainly mediates the automatic inhibition of Ach release. In PD patients, the level of Ach in the striatum increased and cholinergic neurons continuously released Ach, which did not seem to be inhibited by M4 receptor feedback. High levels of Ach in the striatum seem to seriously and selectively affect indirect pathway neurons and lead to motor and non-motor symptoms of PD (Sharma et al., 2016).

Adenosine plays a neuroregulatory and homeostasis regulatory role in the CNS. This neuroregulatory effect of adenosine makes it a valuable and effective therapeutic target in various neurodegenerative diseases such as PD, HD, or AD and appears to have benefits in some psychiatric disorders as well.

Numerous neurotransmitters also play a prominent role in gut physiology, affecting gut movement, nutrient absorption, GI innate immune system, and GI flora. The disorder of neurotransmitter levels in PD may be one of the causes of various GI symptoms and other manifestations (Mittal et al., 2017).

#### Endocrine

##### Enteroendocrine Cells

Enteroendocrine cells (EECs) along the GI tract represent were found to be the biggest producers of hormones and biogen amines in the body, remarking the function of histamine, serotonin, and catecholamines. EECs lining the GI tract might also serve as a conduit for the central spread of misfolded α-syn (Chandra et al., 2017).

##### Brain-Gut Peptides

Plenty of brain-gut peptides have neuroprotective effects *in vivo* and *in vitro*, and the mechanisms may be related to anti-inflammation, anti-oxidative stress, anti-apoptosis, neurotrophic action, and autophagy (Zheng et al., 2021). The brain-gut peptides related to the pathogenesis and treatment of PD mainly include GLP-1, ghrelin, nesfatin-1, and pituitary adenylate cyclase-activating polypeptide (PACAP) (Dong et al., 2019). Serum levels of them show varying degrees of reduction in PD patients (Song et al., 2017; Emir et al., 2019; Pham et al., 2022).

The research on GLP-1 is more extensive and comprehensive. Most of the GLP-1 in CNS comes from the periphery and can cross the BBB freely by diffusion, and a few of them are produced by neurons and glial cells (Athauda and Foltynie, 2018). GLP-1 has many biological functions such as anti-inflammation, inhibiting apoptosis, reducing appetite, inhibiting gastric emptying and gut peristalsis, and reducing weight (Yildirim Simsir et al., 2018). The neuroprotective effect of GLP-1 analogs can alleviate the dyskinesia of the PD model, but it can also lead to constipation, abdominal pain, indigestion, anorexia, and other GI adverse reactions (Badawi et al., 2017). Dual GLP-1/GIP receptor agonists (DA) have been tested to perform better than single GLP-1 receptor agonists (exenatide, liraglutide, etc.) in PD mouse models and are considered promising as therapeutic agents for PD. Dual agonists include DA-JC1, DA-JC4, and DA-CH5 (Feng et al., 2018; Zhang et al., 2020). Clinical trials had evidence that exenatide improves dyskinesia in patients with PD (Mulvaney et al., 2020).

Ghrelin may be involved in anti-apoptosis, anti-inflammation, anti-oxidative stress, neurotrophic effect, and autophagy, and has a neuroprotective effect on PD (Bayliss et al., 2016). The reduction in serum levels of ghrelin was more pronounced in patients with PD weight loss (Fiszer et al., 2010). Ghrelin was neuroprotective against neurotoxicity in a variety of PD models. It antagonized neuronal apoptosis and dopamine loss (He et al., 2022). It also inhibits microglial activation, astrocyte-induced inflammatory responses, and oxidative stress (Moon et al., 2009; Jiao et al., 2021). The acylated form of which is essential for the biological activity of ghrelin. Ghrelin or its agonists can also be used to treat GI symptoms that occur with PD and levodopa treatment (Shi et al., 2017).

The neuroprotective effects of Nesfatin-1 may be related to anti-apoptosis, anti-inflammation, and anti-oxidative stress. Nesfatin-1 can antagonize the toxic effects of MPP + on dopaminergic cells by restoring mitochondrial function, inhibiting cytochrome C release, and caspase-3 activation (Dore et al., 2017; Shen et al., 2017). A recent study by Kuo et al. (2021) found that astragaloside IV and Nesfatin-1 encapsulated phosphatidylserine liposomes conjugated with wheat germ agglutinin and leptin activated the anti-apoptotic pathway and blocked the expression of phosphorylated tau protein, promising for the treatment of PD.

Pituitary adenylate cyclase-activating polypeptide exhibits neuroprotective effects in multiple animal models of PD (Maasz et al., 2017; Reglodi et al., 2017, 2018; Hajji et al., 2019). PACAP exerts its neuroprotective effects of antioxidant stress mainly by inhibiting ROS and caspase3 activation by PKA, PKC, and MAPK signaling pathways (Reglodi et al., 2018). PACAP has been shown to enhance the expression of tyrosine hydroxylase (TH) and VMAT2, protect dopaminergic neurons against the neurotoxin 6-OHDA, regulate neuronal mitochondria, and inhibit inflammation. DA neurons in the SN of PACAP knockout mice are more susceptible to paraquat than wild-type mice.

To summarize numerous *in vitro* and *in vivo* studies have shown that four brain-gut peptides GLP-1, PACAP, Nesfatin-1, and Ghrelin exert their neuroprotective effects through similar molecular mechanisms and signaling pathways and ameliorate PD motor symptoms. Most of them play a significant neuroprotective role in PD by inhibiting caspase-3 activation, reducing mitochondria-related oxidative stress, and inhibiting microglial activation and anti-autophagic activity (Zheng et al., 2021). It suggested that there is a close link between GBA dysfunction and neurodegenerative diseases. Analogs of brain-gut peptides are being developed and are undergoing relevant animal and clinical trials as new promising therapeutic strategies for PD (Dong et al., 2019; Glotfelty et al., 2020; Apostol et al., 2022).

### The Immune System

Inflammatory reaction affects neurological control through the GBA, modulating the cooperation between the CNS, ENS, and the gut-associated lymphoid tissue (GALT) (Seguella et al., 2019). CRP, TNF-α, interleukins (IL), and other cytokines can reflect the collective immune-inflammatory state of the body. Mice with traditional microbiota produce sufficient lymphocyte-driven immune response to protect tissue during brain injury (Singh et al., 2018). The regulation of GI flora on immune cells is transmitted to the brain through the migration of T cells from the GI tract to the meninges (Benakis et al., 2016). Gut infection in PINK1 knockout mice triggers an autoimmune mechanism mediated by cytotoxic mitochondrial specific CD8+ T cells, which is related to the damage of dopaminergic neurons (Matheoud et al., 2019).

Parkinson’s disease may be an autoimmune disease, as approximately 40% of patients have autoreactive T cell activated by SNCA peptides (Sulzer et al., 2017). T cell infiltration had been detected in the SN of PD patients, and the fragment of α-syn source recognized by specific T cells was the antigen epitopes, indicating that T cells were activated by α-syn and autoimmunity. The number of CD4Þ T helper cells and B cells in circulation decreased significantly, indicating that the immune function may be impaired. PD Patients have increased levels of peripheral and CNS inflammation and increased GI permeability (Schwiertz et al., 2018; Elfil et al., 2020). Colon biopsies and fecal markers demonstrate elevated levels of inflammatory cytokines in the intestines and blood (Houser et al., 2018; Schwiertz et al., 2018). This suggests that the process of migration of peripheral activated immune cells to the brain directly connects the inflammation of the whole body to the brain (Harms et al., 2018). Patients with GI bowel disease taking anti-TNF-α therapy and individuals being treated with non-steroidal anti-inflammatory drugs (NSAIDs) have a decreased risk for PD (Ascherio and Schwarzschild, 2016), suggesting that anti-inflammatory therapy and immunoregulatory therapy may also play a role in the treatment of PD. But in fact, targeted inflammation inevitably faces the risk of immunosuppression, which can easily lead to opportunistic infections. Therefore, high selectivity must be a prerequisite for this therapy (Pajares et al., 2020).

### Mitochondrial Dysfunction

As a vital organelle of energy production, mitochondria play a crucial role in metabolism and oxidative stress. Mitochondrial dysfunction is considered to be one of the causes of neuronal death in PD (Abeliovich and Gitler, 2016). Generally, α-syn can induce multiple neuronal pathological phenotypes, including nuclear, mitochondrial, endoplasmic reticulum, Golgi, lysosome, and synaptic dysfunction (Wong and Krainc, 2017). In particular, the elimination of damaged mitochondria was directly connected to PD pathogenesis given the central role of PD-related genes PINK1 and Parkin in the cellular process of mitochondrial autophagy (Sliter et al., 2018). Gut microflora disorders can transmit signals to mitochondria, change mitochondrial metabolism, activate immune cells, induce inflammation, and destroy the epithelial barrier (Mottawea et al., 2016).

## Treatment

Various hypotheses and treatments for constipation and other GI disorders in PD, including probiotics, antibiotics, analogs and receptor agonists of brain-gut peptides, radical cure of Hp infection, even vagotomy, have been discussed in the previous article. Other possible treatment options for PD, including dietary intervention, physical activity, GI motility drugs, and microflora transplantation, will be introduced in the following sections.

### Diet and Nutrition

Dietary composition and nutritional status have been proved to be one of the most critical changeable factors including heredity, health status, mode of delivery, and environment (Larroya-García et al., 2019). Diet can influence the immune system by regulating the gut microbiota (Wastyk et al., 2021). As a microbial community regulator, an active diet can transform pro-inflammatory bacteria into anti-inflammatory bacteria, which not only alleviates GI dysfunction but also has the potential to treat various neuropsychiatric diseases (Larroya-García et al., 2019). Dietary intervention is thought to be helpful to prevent motor and non-motor symptoms of PD.

There are three famous dietary patterns in the world: the Eastern diet, the Western diet, and the Mediterranean diet. It is common to conduct a comparative study of the Mediterranean diet and the western diet. The Mediterranean diet is dominated by vegetables, fruits, grains, beans, nuts, olive oil, and healthy fats, while the Western diet is known for its high fat, high protein, high sugar, and low dietary fiber intake. The microbial community in the Mediterranean diet is rich in polysaccharide-degrading bacteria, which use dietary fiber and polysaccharides to ferment to produce SFCAs, which can inhibit inflammation and prevent obesity (Shankar et al., 2017). For western diets with low dietary fiber intake, microbiota use proteins as energy sources that are beneficial to the growth of Gram-negative bacteria, and bacteria that produce SFCAs may decrease, leading to metabolic disorders, GI flora imbalance, dysbiosis, and an increase in LPS (Statovci et al., 2017). This can lead to obesity, systemic inflammation, and damage to the BBB (Kendig et al., 2021). This proves that the GI flora can be shaped to some extent by our dietary patterns. This provides a way to regulate GI flora through diet, regulate GI health, and then affect PD (Shankar et al., 2017; Larroya-García et al., 2019). Some studies confirm this assumption. Adherence to the Mediterranean diet in middle age was inversely associated with the risk of developing PD later in life (Yin et al., 2021). Adherence to the Mediterranean diet was also positively associated with lower prodromal PD in older adults (Maraki et al., 2019).

#### Omega-3 Fatty Acids

Omega-3 polyunsaturated fatty acids (ω-3 PUFAs) have the potential to prevent and treat PD as anti-neuritis agents. Several animal studies in aged animals and neurodegenerative models have demonstrated that dietary phospholipid precursors, such as uridine, ω-3 PUFA, and choline, may increase cephalin, axonal growth, synaptic proteins, dendritic spine formation, and neurotransmission *via* the Kennedy pathway (Joffre et al., 2019). ω-3 PUFA is an important component in the cell membrane, which has three main types: EPA (eicosapentaenoic acid), DHA, and ALA (eicosapentaenoic acid). DHA is the most important ω-3 PUFA in the brain, which can reduce oxidative stress and α-syn accumulation (Dyall, 2015).

Omega-3 polyunsaturated fatty acids has a protective effect on dopaminergic neurons, which may be related to its antioxidant and anti-inflammatory properties. DHA treatment reduces astrocyte proliferation and microglia proliferation in striatum and SNPC (Mori et al., 2018). In animal experiments, it was found that a diet containing both uridine and DHA could prevent rotenone-induced motor and GI dysfunction, reduce α-syn accumulation, colon shortening, T cell infiltration, and delayed GI transport (Perez-Pardo et al., 2018). DHA and uridine both reduced drug-induced rotational behavior in the 6-OHDA rat model, possibly by enhancing dopamine turnover in the remaining neurons, attenuating the loss of dopaminergic neurons and striatal nerve endings caused by 6-OHDA toxicity in rats, and partially restoring dopaminergic neurotransmission to ameliorate motor and cognitive function (Gómez-Soler et al., 2018). Interestingly, an *in vivo* study found that ω-3 PUFA can inhibit microglial activation and dopaminergic damage induced by inhibiting LPS-induced activation of NF-κb (Ji et al., 2012).

Different clinical studies have also shown a certain relationship between ω-3 PUFA and the risk of PD: ω-3 PUFA intake in PD patients was significantly reduced, and high ω-3 PUFA intake was associated with decreased risk of PD (de Lau et al., 2005). PD patients take ω-3 PUFA to improve depressive symptoms, plus vitamin E can also have a good effect on UPDRS and insulin metabolic markers (da Silva et al., 2008). In addition, ω-3 PUFA is an effective way to increase SCFA production, thereby improving GI environmental balance. Because ω-3 PUFA supplements induce the reversible increase of several SCFA-producing bacteria, including Bifidobacterium, Lactobacillus roseus, and Lactobacillus (Watson et al., 2018).

#### Dietary Fibers and Short Chain Fatty Acids

Dietary fiber fermented and degraded by GI microorganisms will produce a large number of SCFAs in the gut (Makki et al., 2018; Dalile et al., 2019). SCFAs can directly affect GI physiology and GI barrier function, keep digestive structure in the best state, and enhance GI peristalsis. SCFAs also play an important role in gene expression and mitochondrial function and can induce the expression of anti-inflammatory cytokines, inhibit the expression of pro-inflammatory cytokines and inflammatory cytokines, regulate adaptive immune tolerance, and regulate the levels of GI hormones and neuropeptides (Mulak, 2018).

Short chain fatty acid is the final product of indigestible carbohydrates fermented by the gut Microbiota. The levels of SCFA-producing bacteria and fecal SCFA in PD patients were lower, while the levels of conditionally pathogenic bacteria and carbohydrate metabolic probiotics were higher (Unger et al., 2016; Wallen et al., 2020). *Prevotellaceace* and *Lachnospiraceae* family members, as well as the genus *Blautia*, *Roseburia*, and *Faecalibacterium*, are all involved in GI mucus formation and short-chain fatty acid (SCFAs) production, and their contents in GI microorganisms in PD patients are reduced (Unger et al., 2016; Vascellari et al., 2020). The reduction of SCFA in PD may affect gut permeability and lead to local and systemic sensitivity to bacterial antigens and endotoxin due to the destruction of GI mucus, which may be an environmental trigger for PD.

Recalling the above, the Mediterranean diet promotes SCFAs production to the extent that it maintains a healthy microbiome that contributes to GI homeostasis. The antioxidant and anti-inflammatory effects of SCFAs reduce the potential risk of PD. At the same time, increasing the intake of plant fiber may also relieve constipation symptoms in PD patients. However, one study has the opposite result. They found that when SCFAs was used to treat PD model mice, it could activate microglia, aggravate inflammation, and then aggravate dyskinesia and lead to more serious constipation in PD mice (Sampson et al., 2016).

#### Vitamins

Numerous studies have found some relationship between the vitamin family and PD. Since oxidative stress and neuroinflammation play an important role in neurodegeneration and PD, the antioxidant properties of vitamins and their biological function of regulating gene expression suggest that vitamins may be an effective adjuvant therapy for PD (Zhao et al., 2019). Appropriate vitamin supplementation can reduce the incidence of PD, delay the development of PD and ameliorate the clinical symptoms of PD patients (Shen, 2015; Zhao et al., 2019; Chong et al., 2021; Marie et al., 2021).

The vitamin level in PD patients is different from that in normal people. Lower vitamin B12 level in PD patients is associated with more serious motor dysfunction, while higher homocysteine level is associated with more serious cognitive decline (Christine et al., 2018). The level of vitamin B3 (nicotinic acid) in the feces of PD patients is low (Christine et al., 2018), but the side effects of nightmares and rashes limit the development of nicotinic acid in treating PD. Lack of 25-OH vitamin D and decreased sunlight exposure was also significantly associated with increased risk of PD (Zhou Z. et al., 2019).

However, some studies do not support the above conjecture. The folic acid level of PD patients is similar to that of normal people, and there is no significant correlation between dietary folic acid and vitamin B12 intake and PD risk (Shen, 2015). Vitamin D supplementation can effectively increase the level of 25-OH vitamin D, protect dopaminergic neurons in SN and prevent PD from deteriorating further (Rimmelzwaan et al., 2016), but it has no significant benefit in improving the motor function of PD patients (Zhou Z. et al., 2019). Vitamin C, ascorbic acid (AA), can even enhance the selectivity and toxicity of 6-OHDA in a mouse model. Its mechanism is mainly to induce the increase of intracellular calcium, destroy calcium homeostasis and induce cell death, which leads to the activation of calpain and mitochondrial damage (Wang et al., 2017). At the same time, there is not enough evidence to support the hypothesis that taking antioxidant vitamins (including vitamin E, vitamin C, and carotenoids) can reduce the risk of PD (Hughes et al., 2016).

#### Dietary Restriction and the Ketogenic Diet

Epidemiological data suggest that excessive energy intake, especially in middle age, increases the risk of stroke and Alzheimer’s and PD later in life. Dietary restriction (DR) has been proved to slow down the onset of age-related diseases and prolong life (Madeo et al., 2019). There are two types of DR programs in the clinic: intermittent fasting (IF) and calorie restriction (Rubovitch et al., 2019). Compared with randomly fed PD mice, fasting mimicking diet (FMD) can increase the levels of BDNF and dopamine, inhibit neuroinflammatory response, and regulate the composition of GI microbiota in PD mice. This results in decreased motor function and less loss of dopaminergic neurons in the SN (Zhou Z. L. et al., 2019).

During fasting, when the glycogen reserve of the liver is exhausted, the liver will convert fatty acids into ketone bodies to serve as life fuel, especially to provide the main energy source for the brain. Ketones can regulate the expression and activity of many proteins and molecules that affect health and aging and stimulate the expression of BDNF genes, which are related to brain health, psychiatric and neurodegenerative diseases (Mattson et al., 2018). In addition, a low-calorie diet can ameliorate glucose regulation, strengthen mitochondrial function, reduce insulin resistance, enhance anti-stress ability, reduce the production of free radicals, activate the internal defense of cell oxidation, inhibit inflammation, stimulate autophagy, and even remove or repair damaged DNA molecules, protect neurons from excitotoxicity degeneration, and promote cell survival (Mattson et al., 2017).

Dietary restriction intervention is considered to ameliorate obesity, hypertension, dyslipidemia, inflammation, and ameliorate mood and cognition (Montefusco et al., 2021). Long-term or regular reduction of calorie intake while maintaining nutrition is a reliable strategy to keep mammals healthy with increasing life expectancy. It is believed that calorie restriction can intervene, reverse or prevent age-related diseases, such as neurodegenerative diseases, age-related cardiovascular diseases, and malignant diseases, such as tumors (Madeo et al., 2019; Montefusco et al., 2021).

At a sufficient degree of ketosis, the direct signal effect of the ketone body can induce the gene expression of antioxidant enzymes, reduce apoptosis through a metabolic state similar to fasting, and theoretically increase neurotransmitters. A ketogenic diet with a high percentage of fat, a low percentage of carbohydrates, protein, and other nutrients that cause nutritional ketosis in PD patients has indeed been found to ameliorate the condition to some extent (Phillips et al., 2018; Choi et al., 2021).

#### Others

Caffeine intake is associated with a reduced risk of PD, especially in men, and later onset in coffee drinkers (Palacios et al., 2012; Socała et al., 2020; Gabbert et al., 2022). Absolute serum concentrations of caffeine and its downstream metabolites are significantly lower in PD than in healthy controls (Fujimaki et al., 2018). The beneficial effects of caffeine are achieved by antagonizing adenosine receptors (AR), interfering with GABA receptors, activating ryanodine receptors (RyRs), reducing pro-inflammatory and increasing anti-inflammatory marker levels to exert anti-inflammatory activity (Rodak et al., 2021; Ishibashi et al., 2022). Therefore coffee can be part of a healthy diet and may even be an adjuvant in the treatment of PD.

Tea consumption may reduce the risk of PD. L-theanine, which is contained in black or green tea beverages, may be a potential therapeutic pathway for PD. It is similar in structure to glutamate, the major excitatory neurotransmitter in the brain, and shows antioxidant and anti-inflammatory properties, increases dopamine supply, ameliorates motor behavior abnormalities, and has neuroprotective effects (Zhou Z. D. et al., 2019; Malar et al., 2020).

High consumption of milk and dairy products was positively associated with the risk of PD (Hughes et al., 2017), possibly related to the effect of dairy products on reducing uric acid (Chiu et al., 2020). Uric acid salts are potent endogenous antioxidants that prevent oxidative stress-induced neuronal degeneration and death. It has neuroprotective properties and may prevent the progression of PD to significant motor symptoms. Low blood uric acid is a risk factor for PD, and this negative association is more pronounced in the male population. In women, urate is protective only at older ages, when urate levels are comparable to those of men (Cortese et al., 2018; Tana et al., 2018).

Alcohol, especially beer intake, is negatively associated with the risk of PD (Zhang et al., 2014), which may be related to increased uric acid levels (Yamamoto et al., 2005). Meanwhile, beer is mostly made by fermentation, while fermented milk intake was not associated with an increased risk of PD (Olsson et al., 2020). The new study found that highly fermented foods steadily increase microbial diversity and reduce inflammatory markers, which could be a potential protective method for PD (Wastyk et al., 2021).

Levodopa is the main drug used to treat PD. A good diet can optimize the desired therapeutic effect of levodopa by facilitating its absorption or reducing its side effects. The effects of dietary fiber, caffeine, and vitamin C mainly depend on improving gastric emptying. Restricting protein intake may promote levodopa absorption, thereby improving clinical efficacy and reducing motor fluctuations. Vitamin B (including vitamin B-12, vitamin B-6, and folic acid) may reduce homocysteine levels and thus reduce metabolic complications caused by levodopa-induced hyperhomocysteinemia (Boelens Keun et al., 2021).

### Physical Activity

Physical exercise can reduce the risk of PD and has a positive impact on the prevention and treatment of PD. It can ameliorate the motor ability and non-motor symptoms of PD patients and is beneficial to cognitive function, mental state, and autonomic nervous function (Gubert et al., 2020). Physical exercise is considered to reduce the accumulation of synaptophysin, regulate neuronal autophagy, inflammation, oxidative stress, reduce neuronal death, enhance mitochondrial function, and increase the activity of BDNF (Fan et al., 2020). At the GI level, exercise can increase key antioxidant enzymes (catalase and GSH peroxidase), anti-inflammatory cytokines, and anti-apoptotic proteins in GI lymphocytes, while reducing pro-inflammatory cytokines and pro-apoptotic proteins, thereby reducing GI inflammation and protecting the nervous system (Allen et al., 2018). Exercise also has a positive effect on GI flora. Moderate exercise can increase the diversity of GI microorganisms and increase the number of bacteria involved in amino acid biosynthesis and carbohydrate/fiber metabolism, thus producing more SCFA and other key metabolites. But excessive exercise may increase inflammation (Clauss et al., 2021). Exercise can reduce constipation, shorten the time of feces in the GI tract, and reduce the contact between pathogens and the mucus layer of the GI tract, thus reducing contact with the circulatory system and absorption of toxins (Allen et al., 2018). Exercise can also produce a state of IF similar to that mentioned above: induce ketosis, and then eat, rest, or sleep (Mattson et al., 2018). Intermittent metabolism is formed in the whole life cycle, which optimizes brain function and flexibility, especially in neuron circuits involving cognition and emotion (Mattson et al., 2018).

### Medicine

Some anti-PD drugs have side effects that can cause GI symptoms. GI damage, in turn, can interfere with drug absorption, creating a negative cycle. For example, the delayed gastric emptying caused by levodopa. The GI tract of PD may have GI motility decline, motor dysfunction, related autonomic nerve dysfunction, and other effects caused by various anticholinergic and dopamine agonist drugs.

Parkinson’s disease patients treated with camicinal (GSK962040), a gastroprokinetic, showed ameliorated motor responses to levodopa (Marrinan et al., 2018). The inhibition of α-syn aggregation and toxicity by squalamine can significantly change the bioavailability of other drugs. Oral ENT-01 (a synthetic squalamine salt) is safe for GI function in PD patients. It can quickly and effectively restore disordered colonic activity and significantly ameliorate GI function, so it may be beneficial to the treatment of constipation (West et al., 2020). This suggests that ENS is not irreversible damage in PD patients, and its improvement may be local stimulation of ENS through Lewy corpuscles, thus alleviating PD-related CNS damage.

The neurodegeneration of PD is accompanied by microglial activation, upregulated expression of cyclooxygenase-1 and -2, increased inflammatory cytokines and related molecules, and the involvement of leucine-rich repeat protein kinase 2 (LRRK2) protein in the inflammatory pathway (San Luciano et al., 2020). In theory, anti-inflammatory drugs can delay or prevent the clinical development of PD, especially LRRK2-Related PD, by inhibiting the cyclooxygenase-2 enzyme and the pro-inflammatory response of microglia (Crotty et al., 2020). NSAIDs aspirin and ibuprofen reduce LRRK2 episodes and lower the risk of PD (Fyfe, 2020). Aspirin has the greatest effect on age at onset, and its total intake, number of weekly doses, and duration of dosing are associated with delayed onset of PD (Gabbert et al., 2022). However, no significant association has been found between the use of other NSAIDs and the risk of PD (Poly et al., 2019), suggesting that aspirin may have a specific neuroprotective effect.

### Fecal Transplantation

Fecal microflora transplantation (FMT) is the most effective method for the intervention of GI microflora, and the recovery of GI microflora to the pre-onset state of PD is a promising method for prevention and treatment (Wang et al., 2021). FMT has been shown to protect PD mice by inhibiting neuroinflammation and reducing TLR4/TNF-α signaling (Sun et al., 2018).

Fecal microflora transplantation plays a positive role in the treatment of PD, and has the potential to reconstruct the gut microbiota of PD patients and ameliorate their motor and non-motor symptoms (Huang et al., 2019; Kuai et al., 2021). It transplants the GI microbiome of a healthy person into the patient’s intestines through nasogastric, nasoduodenal, or rectal enema tubes. FMT can significantly reduce the disorder of GI microflora and supplement GI microflora beneficial bacteria, such as SCFA-producing bacteria. These bacteria can regulate GI function and protect the mucosal barrier (Zhu et al., 2021). FMT can increase the levels of striatal neurotransmitters DA and 5-HT, decrease the activation of microglia and astrocytes in SN, inhibit neuroinflammation and ameliorate motor function (Sun et al., 2018). FMT has a certain prospect in the treatment of PD, but the development of this therapy is limited because of the rapid change of the bacterial community and the short effective time after a single treatment. At the initial stage of transplantation, the microflora structure of the patient was similar to that of the donor, and the leg tremor and constipation were significantly ameliorated.

However, as time goes on, the difference in the structure of GI flora gradually appeared, and the effect of this treatment gradually weakened. This showed that the GI flora status of patients is significantly related to the severity of symptoms (Huang et al., 2019). Two solutions are possible in the future: one is to prolong the duration of the treatment and establish a good GI flora through diet, drugs, and other auxiliary microflora transplantation; the second method is to develop a new method of transplanting fecal flora, so that flora transplantation can be as painless as taking medicine, to the extent that it can be transplanted regularly. FMT also carries the risk of infection transmission, which may cause bacterial infections such as bacterial metastasis, septicemia, and multi-drug resistant organisms (Wang et al., 2021). Given this, we should strengthen the physical examination of donors to ensure the quality of feces. At the same time, additional antibiotics are used within a reasonable range to optimize the cure rate of FMT (Cheng et al., 2019).

## Conclusion

**FIGURE 4:**
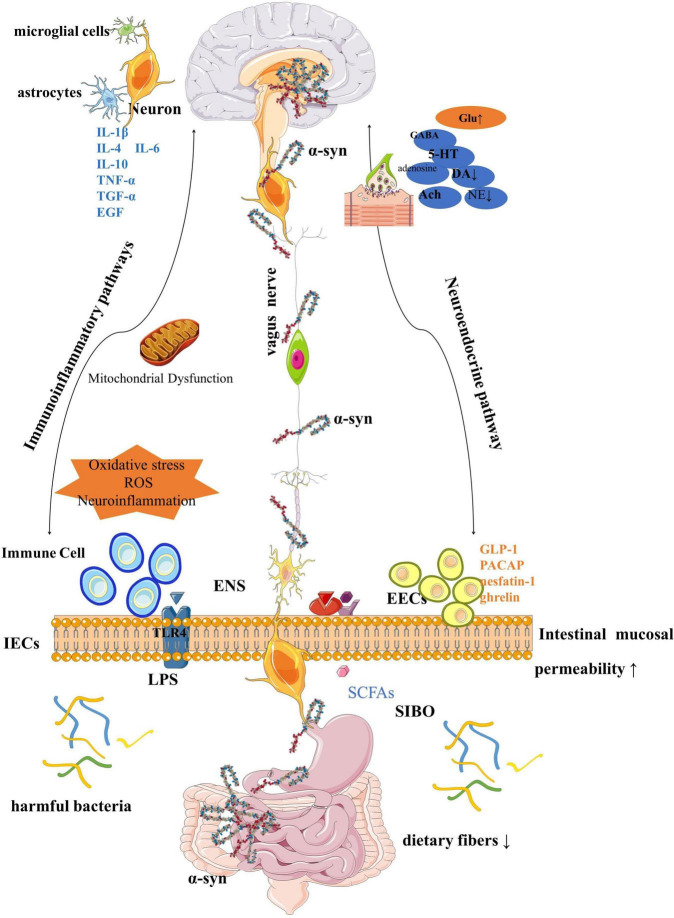
Gut-brain axial pattern diagram.

Although the article is divided into sections for a more coherent description, in fact, each factor is interrelated and inseparable, and together constitutes the homeostasis and balance of the GBA (Figure 4).

For more than 20 years before the diagnosis of PD, the GI tract was disturbed by various factors *in vivo* and *in vitro*, resulting in a variety of gut prodromal pathological changes, such as abnormal accumulation of α-syn, mitochondrial dysfunction, and GI inflammation, through the ANS, crossing the BBB, constantly affecting the functional state of the brain. This is most likely a pathway leading to PD. During this period, various symptoms of the GI tract, especially persistent and painful constipation, constantly remind us of the deterioration of human function. Although GI symptoms are not fatal, these dysfunctions can lead to impaired quality of life in PD patients, affect drug absorption, and cause symptoms fluctuate.

Some animal studies clearly showed that the gut microenvironment can affect behavior through various compounds produced by the gut and its microbes (gut-to-brain), whereas stress can perturb the composition of the microbiota (brain-to-gut) (Schretter et al., 2018; Gubert et al., 2020; Lee et al., 2021).

Based on the GBA theory of PD, we can see many promising ideas and methods for the treatment of PD. Increasing beneficial dietary intakes, such as probiotics, ω-3, SCFAs, vitamins, a Mediterranean diet, a low-calorie intermittent diet, and moderate exercise are all ways to ameliorate GI status and PD. Eradication of *Hp* and brain-gut peptide analogs also have certain therapeutic potential. In addition, FMT to regulate the onset of PD by interfering with GI microflora seems to be an effective treatment.

## Discussion

In our review, we comprehensively described the pathogenesis of GBA in PD, summarized the relevant treatments, and put forward some promising treatments for PD. However, in the process of writing, it is found that there are many different research results of pros and cons, some of which I have given my understanding and explanation, and some may prove that that point of view is still in doubt and needs future generations to continue to study. In addition, the following four suggestions are put forward for follow-up researchers in this field.

We should admit that constipation in PD patients may result not only from myenteric ganglion dysfunction or the mechanisms mentioned above but also from side effects of anti-PD drugs, especially anticholinergic drugs and dopamine agonists (Fasano et al., 2015). Therefore, constipation in PD patients should be recognized in two stages: one is constipation that occurs before the official diagnosis of PD, which is a precursor symptom that can help diagnose PD; the other is constipation that occurs after the diagnosis of medication, which may be caused by the progression of the disease, but the effect of drugs cannot be completely ruled out.

We suggest a multidisciplinary collaborative approach and a greater role for neurology and gastroenterology in untangling the mysterious role of the GBA in various neurodegenerative diseases. At the same time, we have learned that other diseases related to the GBA mechanism of PD include SIBO, IBD, appendicitis, and even systemic diseases such as tuberculosis and diabetes (Chen et al., 2021; Dãnãu et al., 2021; Li et al., 2021; Lv et al., 2022). Small intestinal bacterial overgrowth (SIBO) is very common in PD, and it is hypothesized that the more severe the degree of PD, the more severe the GI motility impairment and the more prone to SIBO (Fasano et al., 2013). It is also thought that it may play a synergistic role with Hp infection in the pathogenesis of PD motor fluctuations. People with IBD are at higher risk of developing PD in later life. They share a common LRRK2 allele, which may indicate that LRRK2 kinase inhibitors also have therapeutic potential (Lee et al., 2021). The appendix is thought to contain aggregates of α-syn, and appendectomy affects the incidence of PD (Killinger et al., 2018; Chen et al., 2021). The risk of PD in tuberculosis patients was 1.38 times higher than that in normal people, and Mycobacterium paratuberculosis was also associated with PD (Shen et al., 2016; Dow, 2021). *In vitro* model, the anti-tuberculosis drug rifampicin has been found to have a neuroprotective effect on PD by inhibiting inflammation and apoptotic autophagy. People who were vaccinated with BCG to prevent tuberculosis showed lower PD disease (Klinger et al., 2021). Patients with diabetes are at increased risk of developing PD, which progresses more rapidly and is more severe (Komici et al., 2021). Therapeutic diabetes drugs, particularly DPP4 inhibitors and/or GLP-1 analogs, may beneficially alter the pathophysiology of PD, reduce the incidence of PD, and improve the functioning of PD patients (Brauer et al., 2020; Wang et al., 2020). More in-depth analysis and research can be carried out on the interaction between these diseases and PD, especially whether the treatment of these diseases is also beneficial to PD.

In dietary therapy, there are a lot of discussions about dietary intervention only comparing the western diet with the Mediterranean diet, but few people pay attention to the role of the oriental diet. However, patients who belong to the oriental diet account for nearly half of the global PD patients, and its impact on the changes of GI flora should not be ignored. We suggest that research on the role of various mainstream diets in the world in the pathogenesis of PD can be carried out.

The correct diagnosis rate of PD has not been significantly ameliorated in the past 20 years. Non-motor symptoms and biomarkers are helpful to ameliorate the early and correct diagnosis of PD. What is described in this paper can potentially ameliorate the early and correct diagnosis of PD, but further studies are warranted to clarify the time and causal relationship between GI microflora and PD, as well as the suitability of microbiome as a biomarker (Scheperjans et al., 2015).

## Author Contributions

XS carried out the literature retrieval, wrote the manuscript, and made the tables and figures. LX critically modified the manuscript. ZW was in charge of statistics. AX funded this project, critically modified the manuscript, and supervised this work. All authors have reviewed and approved the submission and publication of the final manuscript.

## Conflict of Interest

The authors declare that the research was conducted in the absence of any commercial or financial relationships that could be construed as a potential conflict of interest.

## Publisher’s Note

All claims expressed in this article are solely those of the authors and do not necessarily represent those of their affiliated organizations, or those of the publisher, the editors and the reviewers. Any product that may be evaluated in this article, or claim that may be made by its manufacturer, is not guaranteed or endorsed by the publisher.

## Abbreviations

Ach: acetylcholine
AD: Alzheimer’s disease
ANS: autonomic nervous system
BBB: blood-brain barrier
BDNF: brain-derived neurotrophic factor
BM: bowel movement
CBM: complete bowel movement
CNS: central nervous system
DA: dopamine
EECs: enteroendocrine cells
EGF: epidermal growth factor
EGC: enteric glial cells
ENS: enteric nervous system
GABA: γ-aminobutyric acid
GALT: gut-associated lymphoid tissue
GBA: gut-brain axis
GH: growth hormone
GHSR: growth hormone secretion-promoting receptor
GI: gastrointestinal
GP: globus pallidus
GPe: globus pallidus externa
GPi: globus pallidus interna
HD: Huntington’s disease
IECs: intestinal epithelial cells
IL: interleukin
LPS: lipopolysaccharide
LBs: Lewy bodies
LRRK2: leucine-rich repeat kinase 2
PD: Parkinson’s disease
SBM: spontaneous bowel movements
SCFA: short chain fatty acid
SIBO: small intestinal bacterial overgrowth
SN: substantia nigra
SNpc: substantia nigra pars compacta
SNpr: substantia nigra pars reticulate
TFN-α: necrosis factor α
TGF: transforming growth factor
TH: tyrosine hydroxylase
TLR4: toll-like Receptor 4
α -syn: α-synuclein.
